# CD36 polymorphisms and the age of disease onset in patients with pathogenic variants within the mutation cluster region of APC

**DOI:** 10.1186/s13053-021-00183-0

**Published:** 2021-04-29

**Authors:** T Connor, M McPhillips, M Hipwell, A Ziolkowski, C Oldmeadow, M Clapham, PG Pockney, E Lis, T Banasiewicz, A Pławski, RJ Scott

**Affiliations:** 1 School of Biomedical Sciences, Faculty of Health, University of Newcastle, Callaghan Campus, NSW 2308 Newcastle, Australia; 2 Division of Molecular Medicine, NSW Health Pathology North, 2305 New Lambton, NSW Australia; 3 Centre for Clinical Epidemiology and Biostatistics, University of Newcastle, Newcastle, NSW Australia; 4 Department of Surgery, John Hunter Hospital, Newcastle, Australia; 5 Department of General, Endocrinological Surgery and Gastroenterological Oncology, Poznan University of Medical Sciences, Poznan, Poland; 6 Institute of Human Genetics, Polish Academy of Sciences, Poznan, Poland; 7 Hunter Medical Research Institute, John Hunter Hospital, 2305 New Lambton, NSW Australia

**Keywords:** FAP, Disease phenotype, Polyposis, Modifier gene, CD36

## Abstract

**Background:**

Familial adenomatous polyposis (FAP) is an autosomal dominant condition that predisposes patients to colorectal cancer. FAP is the result of a loss of APC function due to germline pathogenic variants disrupting gene expression. Genotype-phenotype correlations are described for FAP. For example attenuated forms of the disease are associated with pathogenic variants at the 5’ and 3’ ends of *APC* whilst severe forms of the disease appear to be linked to variants occurring in the mutation cluster region (MCR) of the gene. Variants occurring in the MCR are phenotypically associated with hundreds to thousands of adenomas carpeting the colon and rectum and patients harbouring changes in this region have a high propensity to develop colorectal cancer. Not all patients who carry pathogenic variants in this region have severe disease which may be a result of environmental factors. Alternatively, phenotypic variation observed in these patients could be due to modifier genes that either promote or inhibit disease expression. Mouse models of FAP have provided several plausible candidate modifier genes, but very few of these have survived scrutiny. One such genetic modifier that appears to be associated with disease expression is *CD36*. We previously reported a weak association between a polymorphism in CD36 and a later age of disease onset on a relatively small FAP patient cohort.

**Methods:**

In the current study, we enlarged the FAP cohort. 395 patients all carrying pathogenic variants in *APC* were tested against three CD36 Single Nucleotide Polymorphisms (SNP)s (rs1049673, rs1761667 rs1984112), to determine if any of them were associated with differences in the age of disease expression.

**Results:**

Overall, there appeared to be a statistically significant difference in the age of disease onset between carriers of the variant rs1984112 and wildtype. Furthermore, test equality of survivor functions for each SNP and mutation group suggested an interaction in the Log Rank, Wilcoxon, and Tarone-Ware methods for rs1049673, rs1761667, and rs1984112, thereby supporting the notion that CD36 modifies disease expression.

**Conclusions:**

This study supports and strengthens our previous findings concerning CD36 and an association with disease onset in FAP, AFAP and FAP-MCR affected individuals. Knowledge about the role CD36 in adenoma development may provide greater insight into the development of colorectal cancer.

## Background

Familial adenomatous polyposis (FAP) is an autosomal dominantly inherited condition affecting between 1 in 7,000–22,000 people [1, 2]. Approximately 20–30 % of these patients will be considered *de novo* presentations and are likely to present with more advanced disease [3]. The clinical diagnosis of FAP is based on the presence of 100’s to 1000’s of colorectal adenomas. Adenoma development can be macroscopically observed in the colon and rectum by the first to second decades of life. Many patients will be asymptomatic and, if adenomas are not detected early and appropriate prophylactic measures taken, develop with almost certainty into colorectal cancer by the age of 50 [4, 5]. Before identifying *APC*, FAP was considered to be relatively restricted to colonic polyposis and or Gardner’s syndrome, with or without the presence of congenital hypertrophy of the retinal pigment epithelium. After the identification of *APC*, the phenotype expanded to include variant forms of the disease, which ranged from very mild disease (reduced expressivity) to allelic variants that did not display overt polyposis [6–11].

FAP results from the reduced or absent expression of *adenomatous polyposis coli* (*APC*) located on chromosome 5q21-22.2 [12]. Overall, there appears to be a genotype-phenotype correlation in patients with FAP (for review, see Nieuwenhuis and Vasen 2006 [13]), with severe disease being associated with variants occurring in the mutation cluster region (MCR) of *APC* [14] and attenuated FAP being linked to variants 5’ of codon 157 [15] and 3’ of codon 1581 [16]. Notwithstanding, there are significant exceptions to the notion of a genotype/phenotype correlation in FAP, which is more readily explained by other factors that impact adenoma multiplicity.

Mouse models of FAP have been used extensively to search for genetic modifiers of disease expression and multiple murine modifier genes have been reported [17–21]. Thus far, modifiers of FAP have been difficult to associate with human disease, with some of the mouse model contenders showing no association in humans [22, 23]. Recently, the Mom5 locus, which encompassed *Cd36*, was identified as a potential modifier of disease expression in a mouse model of FAP [24]. We have previously reported tentative evidence that polymorphisms in *CD36* influence the age at which polyposis expression occurs [25], based on a relatively small population of patients with confirmed *APC* pathogenic variants.

In this study, we present additional evidence that adds integrity to our previous report and confirms that variants in *CD36* influence disease expression, especially in patients who carry germline variants in the mutation cluster region of *APC*.

## Methods

The patient cohort consisted of 432 individuals clinically diagnosed with familial adenomatous polyposis with a pathogenic germline variant in *APC*. Those patients who did not have a genetic diagnosis of disease were excluded from the study. 278 patients were from Australia and 154 from Poland. All patients were ascertained between the years 1997–2017, and their status assigned at the time of diagnosis or their last clinical follow-up.

Patient groups were divided according to the pathogenic variant site within *APC*, which correlated with phenotype: severe (FAP-MCR bounded by codons 1250–1513); attenuated (AFAP = 5’end spanning exons 3 to 5; 3’distal end and those in exon 9); and intermediate (FAP = the rest of the gene).

From the total cohort of 432 there were 37 patients that did not have enough DNA or there was missing clinical information to allow the sample to be included in the analysis. From the remaining 395 patients, there were 147 AFAP patients, 172 FAP patients and 76 patients who had pathogenic variants in the mutation cluster region (MCR-FAP) of *APC.* The clinical data collected for this study included the age of diagnosis of polyposis. They were censored at the time of blood collection for the detection of pathogenic variants in *APC*.

The 395 FAP patient samples were used for genotyping three SNPs in CD36; rs1049673 (C > G), rs1761667 (G > A) and rs1984112 (A > G) located on chromosome 7q21.11. TaqMan SNP assays (Applied Biosystems) were used to identify the respective variants’ presence or absence. SNP rs1049673 is located in exon 15 (3’-UTR), rs1761667 and rs1984112 are intronic variants flanking exon 1 A [26]. The SNP rs1761667 has been shown to reduce protein expression, while 3’-UTR variants often contain regulatory regions that post-translationally influence gene expression [27]. The 3 SNPs are in strong LD with three haplotype blocks described in the HapMap database [28]. Allelic discrimination was undertaken according to the Taq- Man SNP Genotyping Assay Protocol, involving; 10 min at 95 degrees; 40 cycles of 15 s at 95 degrees; and 1 min at 60 degrees. For rs1049673, 407 genotypes were recorded; for rs1761667, 338 genotypes, and rs1984112, 404 were recorded. The reason for the failed genotyping was inadequate amounts of DNA.

Results were read using the ABI 7500 standard real-time PCR system (Applied Biosystems). Raw data were analysed using TaqMan Genotyper Software (Life Sciences, Foster City, CA). A Pearson’s Chi-square test was used with *P*-values were calculated with degrees of freedom from the log-rank, Wilcoxon, and Tarone test, along with the number of subjects at risk to account the differing weights of each calculation. The SNPs were stratified by mutation group and type, a p-value from a joint test of the interaction term between the mutation group and type.

Statistical analysis was performed using Stata v14.0 (StataCorp LP, TX USA) and SAS v9.4 (SAS Institute, Cary, North Carolina, USA). The cohort of patients was divided into 3 patient groups, APC-MCR (76 patients), FAP (172 patients), and AFAP (147).

Pearson’s Chi-square test was used to evaluate deviation from the expected Hardy-Weinberg equilibrium (HWE). Statistical analysis was performed using Stata 12.1 (StataCorp LP, TX USA). We applied Bonferroni correction for multiple testing, resulting in a corrected significance threshold of *p* = 0.0167 (0.05 divided by the 3 SNPs tested). Due to the nature of the disease and the strong recommendation for prophylactic surgery, the diagnosis of polyposis was used as an endpoint for analysis. Variation in the age of polyposis diagnosis between each SNP; wildtype genotype (homozygous for wildtype allele), heterozygote, and variant genotype (homozygous for variant allele) and mutation group (based on mutation location as described above); was examined using Kaplan-Meier plots. Individuals free from polyposis were censored at their age, at last, follow up. Wilcoxon’s (Breslow), Log-rank, and Tarone-Ware tests were used to examine the Kaplan-Meier plots’ homogeneity. The log-rank test is more sensitive to differences later in time due to equal weighting over the curve, where the Wilcoxon weights the early differences higher than the later differences using the number at risk in the weighting. The Tarone-ware test uses the square root of the number at risk in the weighting. All three tests were required to be significant for results to be considered reliable. Cox regression models were used to provide a formal Wald-test of interaction (global interaction test) between APC mutation groups and SNPs genotypes, taking into account family ID as a group variable.

## Results

The total number of patients recruited into this study was 432 of which 37 could not be included due to either insufficient DNA or the absence of clinical information about the patient. The descriptive statistics outlining age, mutation group, adenomas, and SNPs tested within the population is presented in Table 1.

**Table 1:** Demographics and CD36 data obtained for combined cohort

	Category	Total (***N***=432)
Age	mean (SD)	29.4 (15.3)
	median (min, max)	27 (2, 83)
Mutation group	Other (FAP or AFAP)	319 (81%)
	APC MCR	76 (19%)
	Missing	37
Polyps	No	88 (20%)
	Yes	339 (78%)
rs1049673	Wildtype	141 (32%)
	Heterozygote	184 (43%)
	Variant	82 (19%)
	Missing	25 (6%)
rs1761667	Wildtype	95 (22%)
	Heterozygote	158 (37%)
	Variant	85 (20%)
	Missing	94 (21%)
rs1984112	Wildtype	179 (41%)
	Heterozygote	168 (39%)
	Variant	57 (13%)
	Missing	28 (6%)

The first approach taken was to determine if there was any indication of a difference between the three groups of patients and one or more of the CD36 SNPs under investigation, the results of which are presented in Table 2.

**Table 2:** Test equality of survivor functions for each SNP and mutation group, p-value’s (Chi-squared test statistic, degrees of freedom)

SNP	Log_Rank	Wilcoxon	Tarone	SNP/ Mutation group Interaction
rs1049673	<.0001 (32.89, df=5)	<.0001 (30.73, df=5)	<.0001 (32.11, df=5)	0.0528
rs1761667	<.0001 (26.20, df=5)	<.0001 (25.66, df=5)	<.0001 (26.46, df=5)	0.1917
rs1984112	<.0001 (47.71, df=5)	<.0001 (50.81, df=5)	<.0001 (49.81, df=5)	0.0417

This table represents all three SNPs showing a statistically significant difference at 5 % compared with Wildtype, Heterozygote, or variant mutation groups. For SNP rs1984112, the SNP/Mutation interaction is p = 0.04 indicating a statistical difference between mutation interaction.

**Fig. 1:**
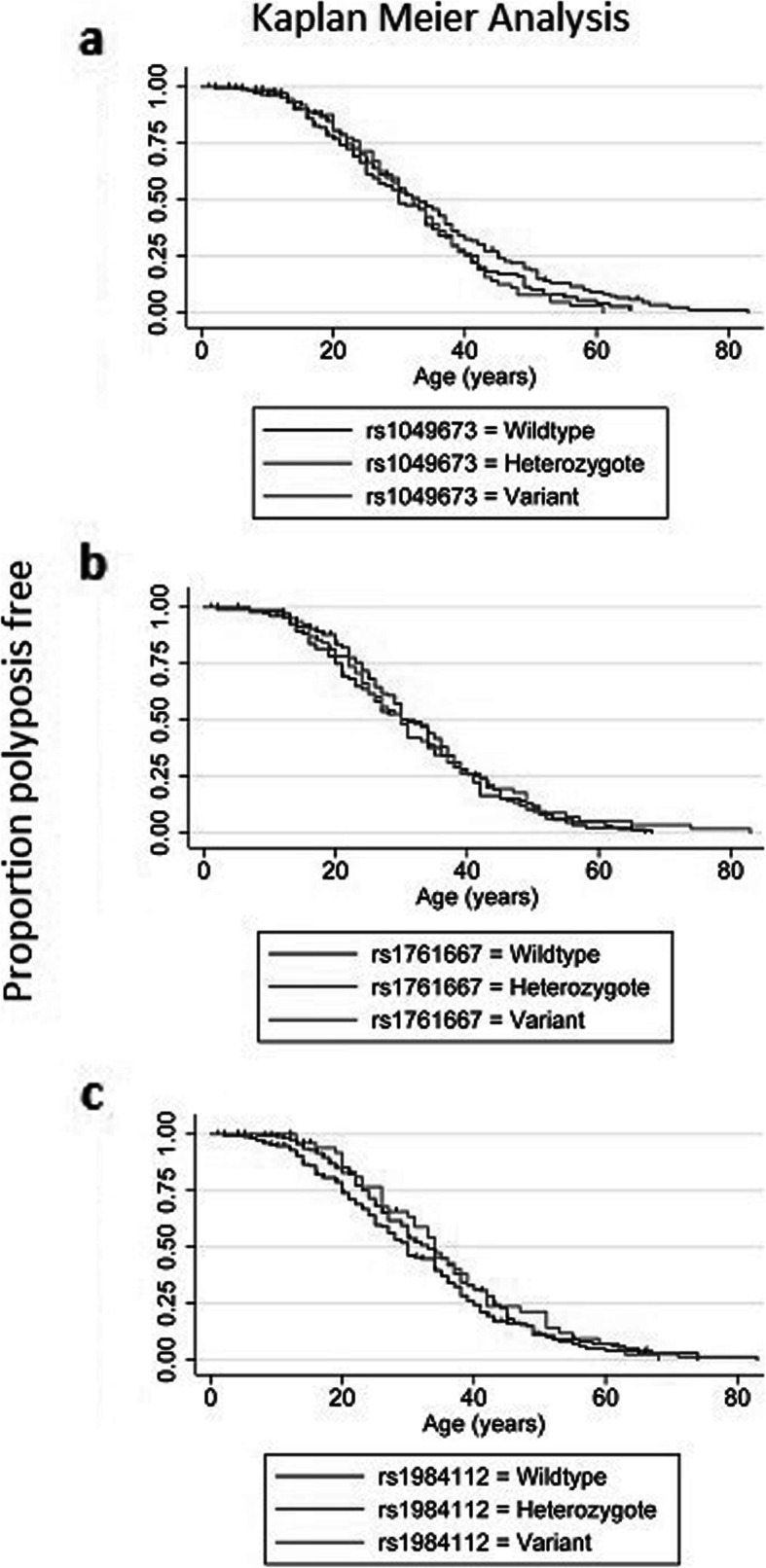
SNP distribution within the entire cohort of patients carrying deleterious APC variants. Overall rs1049673 and rs176667 did not reveal any association with the age of disease presentation. For rs1984112 there appeared to be some evidence that the variant SNP was associated with a slightly later age of disease presentation compared to its wildtype counterpart

For rs1049673, there was no difference in the age of disease diagnosis if the patient was wildtype, heterozygous, or a homozygous variant for this SNP (Fig. 1a). Similarly, for rs1761667, no differences could be observed (Fig. 1b). No statistically significant result was revealed for rs1984112. Still, patients carrying only wildtype alleles did appear to present with the disease at a slightly younger age than heterozygote or homozygote variant carriers (Fig. 1c).

The potential difference between patients homozygous for rs1984112 suggested some effect of this variant on disease expression. Since an analysis of all FAP patients as a single group is not representative of individuals’ phenotypic status, the patients were categorised into three groups based on the location of each patient’s *APC* pathogenic variant and correlated disease severity. Patients carrying variants in those regions of *APC* associated with an attenuated form of the disease were classed as one group (AFAP), patients having pathogenic variants within the MCR were classed as the most severe group (MCR-FAP) and all other patients were considered as “standard” polyposis patients (FAP).

**Fig. 2:**
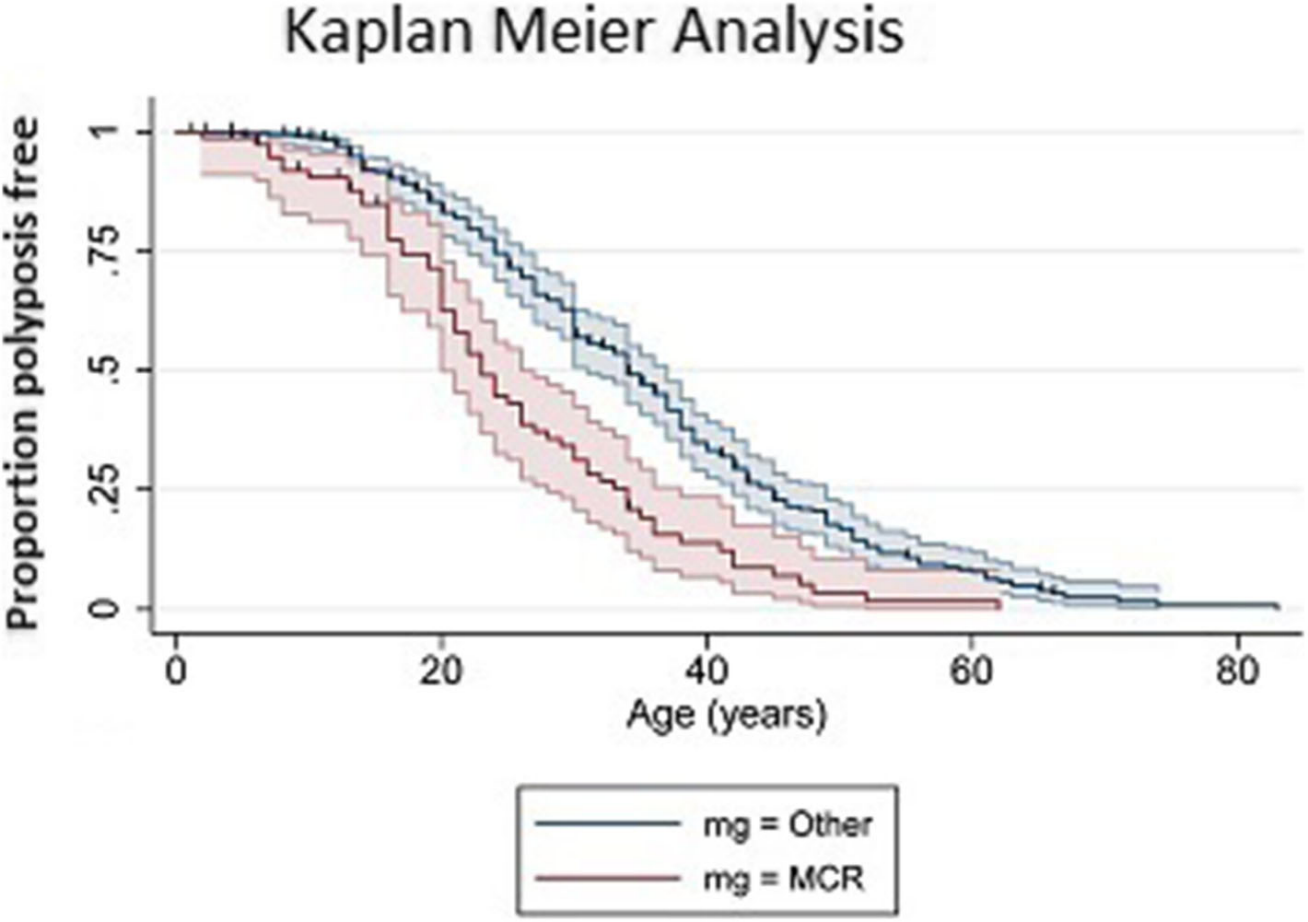
Differences in the average age of disease presentation between patients with APC pathogenic variants bounded within the mutation cluster region (MCR) compared to patients whose germline pathogenic variants resided outside of the MCR

An analysis of the age of disease diagnosis was undertaken focussing on those patients who carried pathogenic *APC* variants in the MCR compared to all other patients (AFAP/FAP). The results revealed that the MCR-FAP group developed the disease approximately 10 years earlier than their AFAP/FAP counterparts (Fig. 2).

**Fig. 3:**
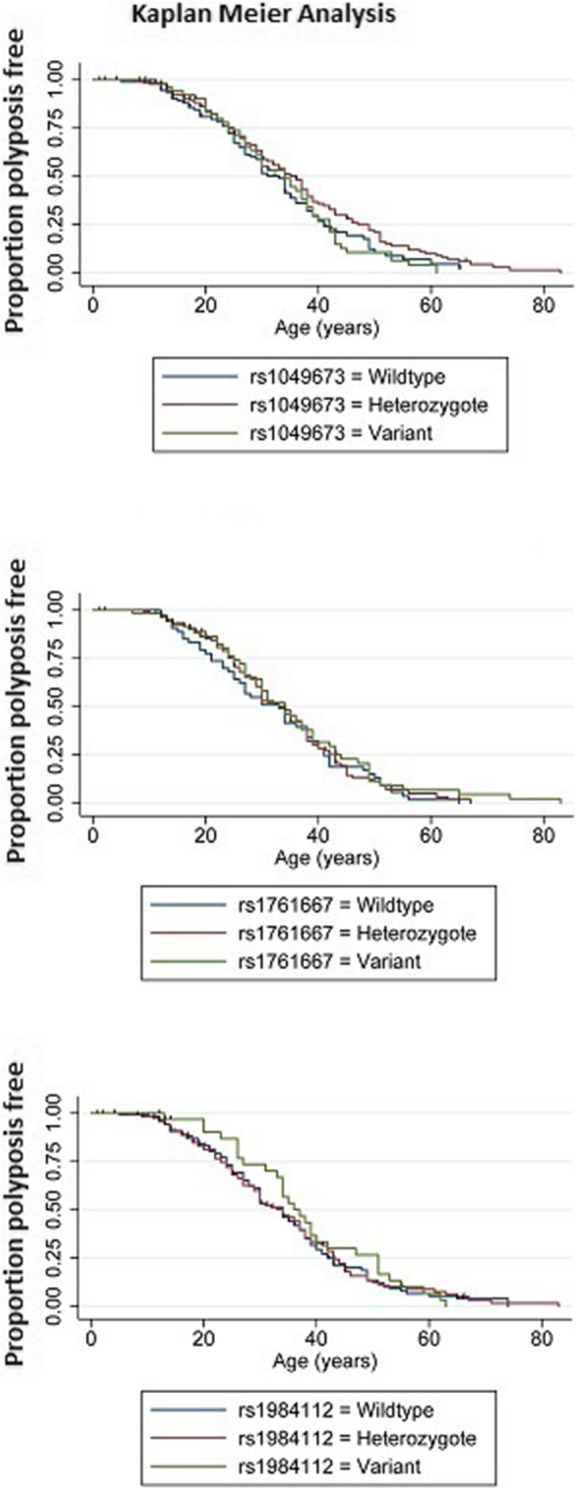
Kaplan Meier analysis of patients carrying pathogenic variants outside of the MCR. Overall, the results were similar for rs1049673 and rs176667 compared to the overall results presented in Fig. 1. For rs1984112 there is some evidence that indicates homozygote variant carriers present with disease at older ages compared to their wildtype and heterozygous counterparts

Examination of rs1049673 revealed no influence of this SNP on the age of disease diagnosis for the MCR-FAP group or the remaining patients even though there remained a difference in the overall age of diagnosis between the MCR-FAP group and the FAP/AFAP group (see Fig. 3a). A similar result was observed for rs1761667 in that this SNP did not influence the age of disease diagnosis (Fig. 3b).

**Fig. 4:**
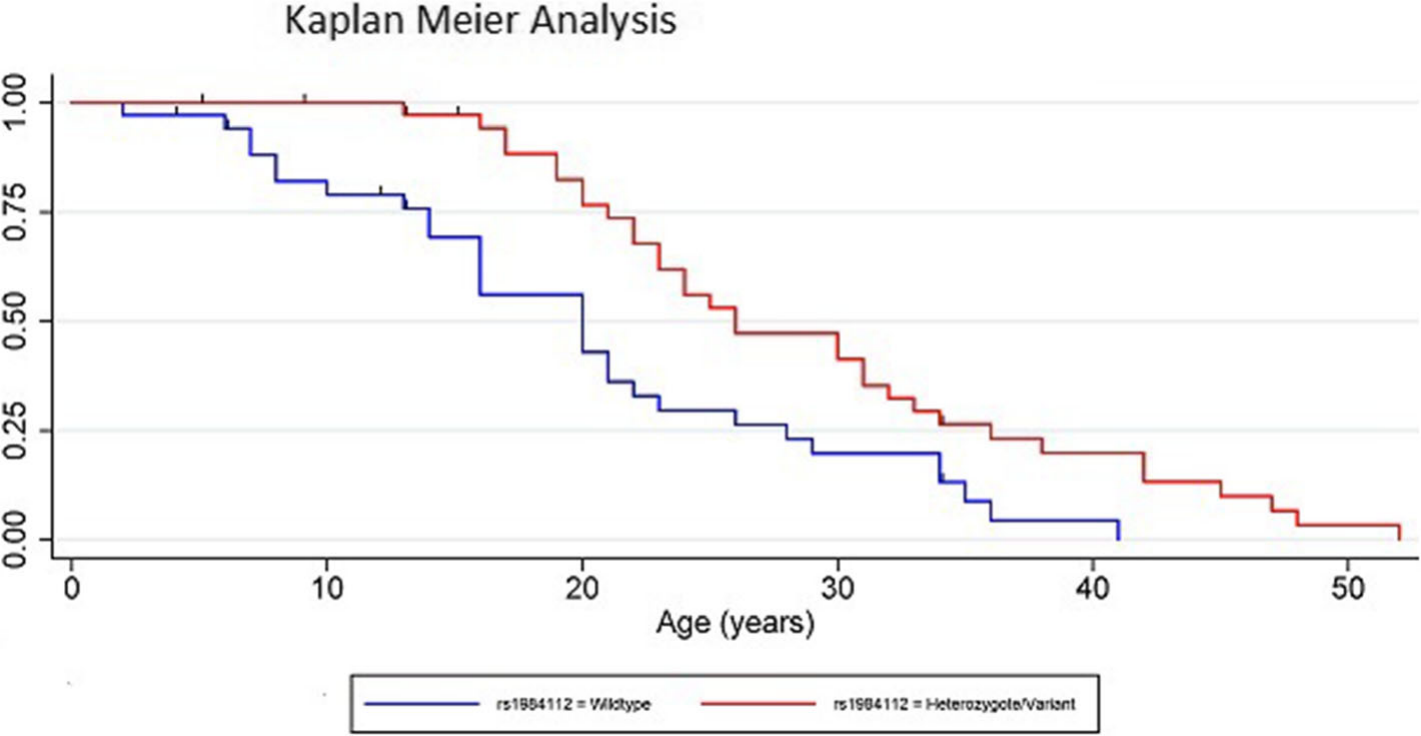
Kaplan Meier analysis for rs1984112 modelling the variant allele as an autosomal dominant modifier. The difference is highly statistically significant for all aspects of the analysis

The three genotypes of rs1984112 were found to be similar in the AFAP/FAP group but significantly different in the MCR-FAP group of patients. Patients who were wildtype for the SNP were considerably younger at the time of diagnosis than patients who carried a wildtype allele and a variant allele or those who had two variant alleles (Fig. 3c). Using a dominant model, when combined, the heterozygote carriers and the homozygote variant carriers were significantly older at the time of their diagnosis compared to patients who were wildtype for this SNP (Fig. 4). When the variant allele’s presence was considered an autosomal dominant modifier, a significant difference between the ages of polyposis presentation was observed, indicating the modifying effects of CD36 resulted in a later age of disease presentation within the MCR-FAP group.

Given the phenotypic heterogeneity in the AFAP group, a comparison between this group and the FAP was undertaken to consider the rs1049673, rs1761667, and rs1984112. No statistical differences in the ages of disease presentations were observed between the AFAP and FAP groups for any SNPs under investigation (data not shown).

Since AFAP has a much milder disease course than FAP, we re-analysed the data focussing on only the MCR-FAP patients and comparing them to FAP patients or AFAP patients alone. Similarly, for rs1409673 and rs1761667, no differences were observed between the three groups (wildtype, heterozygote, and homozygote variant carriers). For rs1984112 wildtype carriers, a statistically significant difference was observed between the MCR-FAP group and the FAP group. When examining the differences between the MCR-FAP and the FAP group, the results indicated a difference in the age of disease onset of all MCR-FAP patients compared to the FAP group. The effect of rs1984112 on disease onset was statistically significantly different in the MCR-FAP group but not the FAP group. A similar finding was revealed when the MCR-FAP group was compared to the AFAP cohort.

## Discussion

Disease expression in FAP is associated with the site of the pathogenic variant residing in APC. It has been extensively reported that a genotype-phenotype correlation exists, which is linked to the location of the pathogenic variant in *APC* and how this influences disease expression [29] via several different mechanisms [30]. Notwithstanding, the correlations that have been described represent approximations that cannot be used to accurately define or predict disease expression in any given patient [31]. An alternative and potentially complementary explanation for the diversity in disease expression observed in FAP is the influence of other factors that could either promote or inhibit disease expression. The presence of one or more modifier genes that influence disease risk in FAP has been reported several times [32, 33], but none have been replicated, thereby casting doubt on their integrity.

Identifying genetic factors associated with phenotypic variability in FAP is the heterogeneity of disease expression related to the location of the causative *APC* pathogenic variant. In this study, we have based our associations on the genotype-phenotype correlations in APC, thereby circumventing the requirement of obtaining actual polyp counts in each patient and the necessity of identifying a large pool of patients all carrying the same pathogenic variant.

Searching for modifier genes in a rare human genetic disorder is challenging, and much focus has been placed on studies of mouse models of disease. The use of the Min mouse (and other mouse models of disease) has revealed several candidate modifier genes that appear to influence disease in the mouse. A relatively recent modifier of Fap, known as Mom-5, was reported in 2015 [24], which seemed to affect adenoma multiplicity. We have previously reported on the role of CD36 in a relatively small group of FAP patients, which revealed a potential association of the wildtype allele of rs1984112 with an earlier age of disease onset compared to heterozygous and homozygous variant carriers [25]. The link between colorectal cancer risk and CD36 has been explored in that variants in rs1984112 have been associated with hypercholesterolemia [34], a known risk factor colorectal cancer [35].

The number of patients in the study by Holmes et al. [25] was too small to interrogate further the relationship between rs1984112 and patients diagnosed with AFAP, FAP, and MCR-FAP. In the current study, it became evident that rs1984112 did not influence the age of disease diagnosis in patients deemed to be AFAP or “standard” FAP. The absence of any effect of rs1984112 on these two groups of patients may be due to the variance in disease penetrance across the cohort [35]. The MCR-FAP group is considered the most severe group of patients with relatively consistent disease expression. It has been well recognised that disease penetrance is not uniform. Still, the MCR-FAP group tends to develop hundreds to thousands of adenomas, which confers a high risk of malignant transformation.

The finding that patients who are wild type for the CD36 polymorphism develop disease somewhere between 5 and 10 years before heterozygotes and homozygote variants is important since this information could be used if replicated to tailor when colectomy be considered. Since adenoma multiplicity is almost impossible to obtain, it is unknown if CD36 polymorphisms can be correlated with adenoma numbers.

## Conclusions

If the findings of this study can be independently verified, FAP-MCR positive patients harboring wildtype polymorphism for rs1984112 may have a 5–10 year earlier onset of polyposis and additional disease risk then heterozygotes and homozygotes. This finding could have a risk stratifying effect on the treatment of disease in MCR affected individuals and enhance our understanding of the disease process’s potential modifier.

## Data Availability

All data generated or analysed during this study are included in this published article.

## Abbreviations

AFAP: Attenuated familial adenomatous polyposis
APC: Adenomatous polyposis coli
CD36: Cluster of differentiation 36
CRC: Colorectal cancer
FAP: Familial adenomatous polyposis
MCR: Mutation cluster region
Min: Mouse intestinal neoplasia
Mom: Modifier of min
SNPs: Single nucleotide polymorphisms
